# Effect of Gradient Heat Treatment on Microstructure and Properties of Cu–Al–Mn Shape Memory Alloy

**DOI:** 10.3390/ma12162505

**Published:** 2019-08-07

**Authors:** Luohui Zhou, Jingling Lan, Jili Liu, Xu Li, Bowen Shi, Shuyang Zheng

**Affiliations:** Hubei Key Laboratory of Theory and Application of Advanced Materials Mechanics, Wuhan University of Technology, Wuhan 430070, China

**Keywords:** gradient heat treatment, Cu–Al–Mn shape memory alloy, graded microstructure, hardness, superelasticity

## Abstract

The columnar-grained Cu–Al–Mn shape memory alloys (SMAs), which have good shape memory properties and are prepared by a unidirectional solidification technique, were subjected to a gradient heat treatment under temperatures ranging from 100 to 450 °C. After this treatment, the microstructure, hardness, transformation temperature and shape memory properties of these samples could exhibit gradient changing trends, all of which were investigated by optical microscope, scanning electron microscopy (SEM), a Vickers microhardness tester, and a compression machine. The microstructure observation result shows that the acicular bainite-precipitated phase produces from scratch and then grows continuously with the increasing of the heat treatment temperature, finally presenting a graded distribution from one end section to another of the sample. The hardness tests give the samples results also increasing with temperature. Specifically, the change relationship between hardness and the treatment temperature mathematically satisfies dynamic function. In addition, it can be concluded from mechanical tests the compressive elastic–superelastic strain and strength of the samples show gradient variation features. Overall, our experimental investigation indicates that a gradient heat treatment is an effective way to conduct microstructure control or design for the Cu–Al–Mn SMAs, and their graded properties are mainly caused by the different fractions of the bainite phase producing in different local areas after the gradient heat treatment.

## 1. Introduction

Shape memory alloys (SMAs) possess intrinsic characteristics, particularly superelasticity and shape memory effect due to a reversible diffusionless martensitic transformation induced by external stimuli such as thermal and mechanical loadings [1,2]. Due to these features, SMAs have strong capabilities as things such as sensors, detectors, dampers, energy converters and smart micro-devices, with a wide range of applications in many fields such as medical and health, mechanical manufacturing, aerospace, civil architecture, and daily life. In recent decades, Cu–A1–Mn SMAs have gradually become one of the key alloys in connection with research and industry because of their low price (equivalent to 1/15 of the Ni–Ti SMA), good conductivity, thermal conductivity, excellent plasticity and workability [3,4,5,6,7,8]. As a kind of innovative SMA with high performance, they attract more and more attention from people.

If the SMA products encounter some harsh service environments, e.g., when designed for foldable or flexible functionality, the ordinary alloys with homogeneous structure and properties can hardly meet the integrated applications standard of SMAs function [9]. An effective method is to make functional gradient alloys, which can adapt to different property requirements or achieve some special aims or combinations. Different from composite materials, functional gradient materials can effectively reduce the mutation of properties caused by the interface mutation, overcome mismatch limitations related to different bonding parts, and improve the comprehensive performance of materials [10].

At present, a functional gradient in an SMA single material can be obtained by a chemical composition gradient, a geometric size gradient and a microstructure gradient [9]. (i) A chemical composition gradient is the most common way. Due to phase transformation temperature, shape memory properties strength, and other properties of SMAs closely related to their chemical composition, SMAs with the graded composition can express the gradient properties. A chemical composition gradient in a certain direction can be achieved by some special methods such as thermal diffusion [11,12], gradient powder forming [13], and additive manufacturing [14]. These methods can be better used to prepare binary graded Ni–Ti alloys, but it is difficult to accurately control the graded composition in ternary and multicomponent alloys such as Cu-based SMAs. (ii) A geometric size gradient means that the gradient change of bearing capacity can be realized in a certain direction after the gradient change design of the geometric section of the alloy, resulting in graded properties [15,16,17]. However, in essence, the gradient achieved by this geometric size gradient is not the gradient of material properties but the gradient of material bearing capacity due to the change of section size. (iii) A microstructure gradient is based on the fact that some microstructures (such as grain orientation, grain size, precipitated phase, and material defects) significantly affect the properties of alloys. Some special methods, such as thermo–mechanical treatments [18,19], graded pore sintering [20], laser surface annealing [21], gradient heat treatments [22] and surface mechanical grinding [23,24], are used to make the microstructure of the alloy gradient change in a certain direction to realize the macro graded properties. This method does not need to change the composition and size of the alloys. It can be used for the functionally gradient preparation of more than three-component Cu-based SMAs.

Sutou and Liu [25,26] also found that the sheet bainite phase (six layered monoclinic 6M structure) precipitated in a Cu–Al–Mn SMA heated at 200~450 °C, and the content of bainite increased exponentially with the increase of heat treatment temperature and time. At the same time, the hardness and tensile strength of the alloy can be significantly improved by the bainite phase, thereby obtaining a high-strength, high-elasticity alloy and achieving adjustable performances in wide range. In this paper, based on the aging precipitation strengthening mechanism of the Cu–Al–Mn SMA, the functional gradient of this alloy was achieved through the microstructure gradient to improve the application performance of the Cu–Al–Mn SMA. The gradient heat treatment method was used to make the structure and properties of the samples presenting gradient evolution. After the gradient heat treatment, the changed distributions of the structure and mechanical properties within the alloys samples were collaboratively studied. The relationship between the graded structure and properties was also investigated, and this can provide important references for the gradient treatment and practical application of Cu–Al–Mn alloys.

## 2. Experimental Materials and Methods

The Cu–Al–Mn SMA ingots for the gradient heat treatment were prepared by unidirectional solidification technology. The specific preparation process is referred to in the literature [27,28]. The chemical composition was Cu with 8.7 wt.% Al and 10.8 wt.% Mn.

**Figure 1:**
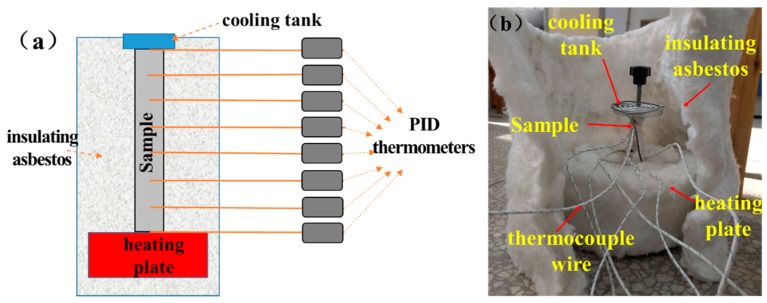
(**a**) Diagram of the gradient heat treatment experiment; (**b**) photo of the heat treatment experiment.

Three samples with the dimension of 70 mm × 6 mm × 4 mm—denoted as #1, #2 and #3 for the gradient heat treatment experiment—were cut by wire electrical discharge machining. K-type thermocouple wires connected with proportion-integration-differentiation (PID) thermometers were separately fixed at 8 positions every 10 mm in the length direction of the samples. The samples were placed vertically on a heating plate (setting temperature was 450 °C, holding for 60 min), and an aluminum tank with water was fixed on the top of the samples for heat dissipation. The heating plate and the rest of the sample were separated and insulated by insulating asbestos. The temperature values of different points on the samples were recorded. The heating schematic diagram and the experimental diagram are shown in Figure 1a,b.

After the gradient heat treatment, the samples were water-cooled, and then were polished and eroded on the metallographic polishing machine (SHOIF P-2). The microstructure of the whole sample was observed by an optical microscope (OM, SHOIF 102 XB-PC) and a scanning electron microscope (SEM, Zeiss Auriga SUPRATM55) after being eroded with an aqueous solution of ferric chloride and hydrochloric acid (5 g FeCl_3_, 10 mL HCl, and 100 mL H_2_O). Then, the microhardness of the heated samples, along the longitudinal section, was measured by a Vickers microhardness tester (HV-1000B). The loading force of the indenter was 200 g, and the holding time was 15 s. Each distance was measured at 8 points in the direction of the width of the samples. The phase transformation temperatures of #1 after the gradient heat treatment, namely the martensite starting temperature *M*_s_, the martensite finishing temperature *M*_f_, the austenite starting temperature *A*_s_, and the austenite finishing temperature *A*_f_, were measured by using differential scanning calorimetry (DSC, Mettler-Toledo DSC 3). The heated #2 sample was compressed to a certain strain along the width, then unloaded, and finally heated at 100 °C; the change of width value along the length of the sample was measured with micrometer. The heated #3 sample was chosen to be evenly cut into nine pieces denoted #3-1–#3-9 from low temperature to high temperature. The mechanical properties of each small block (7.5 mm × 4 mm × 4 mm) under compression cyclic loading and unloading (the force direction along the sample length) were tested by a computer controlled electronic universal testing machine (Instron 5882) with a loading rate of 5 × 10^−4^ s^−1^.

## 3. Results and Discussion

**Figure 2:**
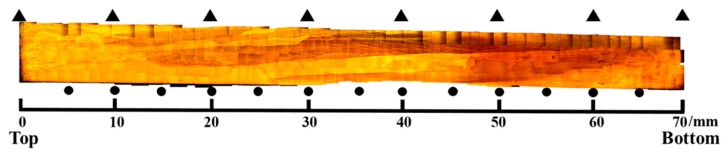
Optical microscope photo of the #1 sample after the gradient heat treatment. ▲ shows the points of temperature measurement. ● shows the points of hardness measurement.

**Figure 3:**
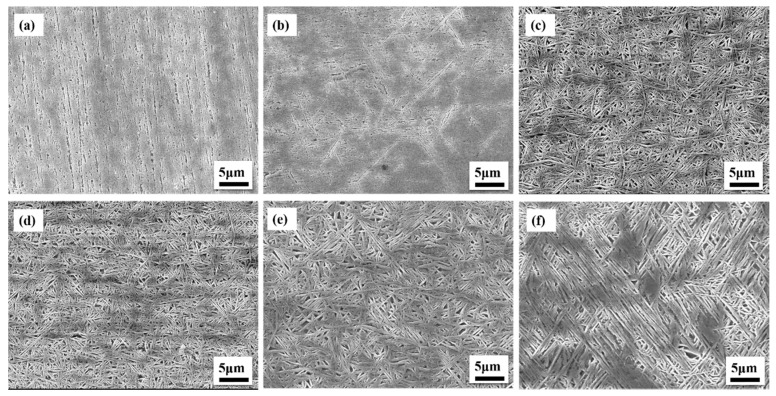
SEM photos of different regions along the length of the #1 sample: (**a**) 160 °C, 10 mm from the top; (**b**) 240 °C, 47 mm from the top; (**c**) 270 °C, 53 mm from the top; (**d**) 300 °C, 64 mm from the top; (**e**) 370 °C, 67 mm from the top; and (**f**) 440 °C, 69 mm from the top.

The OM photo of the Cu–Al–Mn alloy sample is shown in Figure 2. It can be seen that the alloy prepared by unidirectional solidification had high length–diameter ratio grains, which means it had a columnar-grained structure. The microstructure characteristics and property improvement mechanism of the columnar-grained structure Cu–Al–Mn SMA were studied in some previous research [4,28]. In this paper, the matrix structure of the alloy sample was austenite at room temperature. After the gradient heat treatment, moving from the low temperature end to the high temperature end (Figure 2, from left to right, corresponding to the upper end to the lower end of the sample), the color of the sample became darker, which indicates that precipitates appeared at the end of the dark region. Though the morphology of the plate-like precipitates was slightly similar to that of martensite plates, these precipitates formed by agingwere not martensite phase, because the martensitic transformation starting temperature is below the room temperature. According to the literature [25,26], the precipitated phase of the alloy is acicular bainite in the temperature range of the gradient heat treatment in this paper, and a nearly coherent relationship of <022>L21//<006>6M exists between the bainite phase and the austenite phase [26]. The SEM photos of different regions in the sample are shown in Figure 3. It can be seen that the sample showed a graded distribution in different regions. In the region with the temperature below 200 °C, the alloy was the austenite matrix phase; in the region with the heat treatment temperature below 240 °C, a small amount of precipitated phase had begun to precipitate; then, more acicular bainite precipitates showed at 270 °C; as the temperature continued to increase to 440 °C, the number and size of precipitates increased. In the whole length direction of the sample, with the increasing of the heat treatment temperature, the bainite precipitated phase was produced from scratch and then grew continuously (the diameter increasing from 0.1 to about 0.5 micron). The volume fraction of the bainite phase increased gradually with increasing aging temperature. On the whole, the structure of the sample presented a graded distribution from one end section to another.

**Figure 4:**
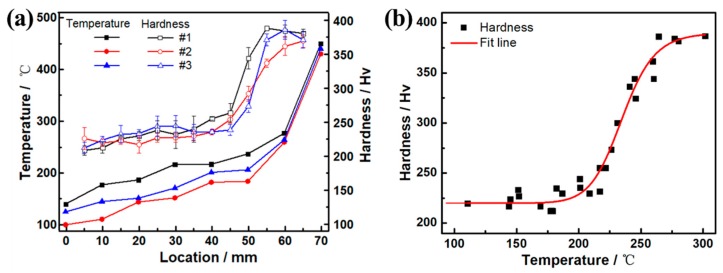
(**a**) Changes of Vickers hardness and temperature with the location along the sample length; (**b**) changes of Vickers hardness with the temperature in the samples.

Because the properties of the Cu–Al–Mn SMAs have great microstructure sensitivity, the microstructure with gradient change will inevitably produce uneven property distribution. The relationships of the Vickers hardness and heat treatment temperature along the sample length are shown in Figure 4a. As the Figure 4a shows, the temperature of the top of sample was between 100 and 140 °C due to contacting with the cooling water tank, and the bottom contacted with the heating platform whose temperature was close to the setting 450 °C. As the sample length changed from top to bottom, the temperature on the three samples continuously rose from 100~140 to about 440 °C. In addition, the rate of temperature change was first slow and then fast. When the region of the sample was close to the end of the heating platform, the rate of temperature change was the fastest. As the continuous gradient of temperature increased, the Vickers hardness value on the sample also increased slowly and then rapidly. The hardness value gradually increased from about 215 Hv at 5 mm from the top of the sample to about 250 Hv at 40 mm, and then it rapidly increased to 388 Hv at 5–10 mm from the bottom. The temperature and hardness distributions of the #1, #2 and #3 samples were different to some extent due to the differences in the tightness between the samples and the platform, as well as the heat dissipation from the external environment. However, the overall trend and law of temperature and hardness changes were consistent. It can be noted from the above analysis that there was a certain relationship between the hardness change and the temperature change of the samples. In this paper, the temperature and hardness of the three samples in different regions are plotted as scatter plots, as shown in Figure 4b. As the temperature of the sample increased, the corresponding values of hardness increased. Combined with microstructural analysis, the hardness dramatically increased with the augment in the number of bainite precipitates and their overlapping, and it gradually became saturated near the end of precipitation. Thus, the increase in hardness was due to the formation of acicular bainite possessing the six layered monoclinic structure, described as 6M. It has been found in literature that the change of hardness with temperature conforms to the dynamic exponential relationship [21]. Therefore, the logistic function commonly used in dynamics was used for fitting in this paper, with a fitting curve of the *VH* = 390 − 170/[1 + (*T*/235)^18^]. As shown in Figure 4b within line, the fitting curve could fit the hardness *VH* change with temperature *T*.

**Figure 5:**
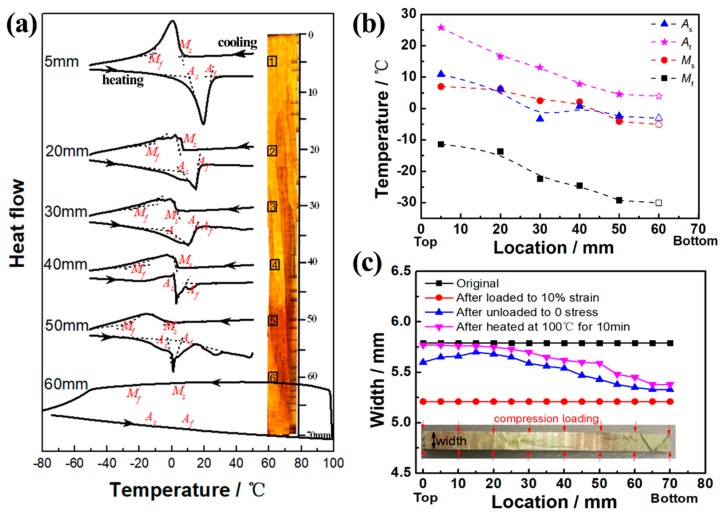
(**a**) Differential scanning calorimetry curves of different regions along the length of the #1 sample. (**b**) Changes of transformation temperatures with the location along the length of the #1 sample. (**c**) The original width, the width after being compression loaded, the width after being unloaded, and the width after being heated with the location along the length of the #2 sample.

Figure 5a shows the DSC cooling and heating curves of different regions along the length of the #1 sample. It can be seen that the exothermic and endothermic peaks—which correspond to martensitic forward and reverse transformations, respectively—decreased and became indistinct with length changes from top to bottom, corresponding to the temperature of the heat treatment increase. The martensitic transformation starting (*M*_s_) and finishing (*M*_f_) temperatures and the reverse transformation starting (*A*_s_) and finishing (*A*_f_) temperatures were defined as the temperatures at which the extrapolation lines of those peaks and the baseline crossed, as shown in Figure 5b. It can be seen that the *M*_s_, *M*_f_, *A*_s_ and *A*_f_ at the top of the sample were 7.0, −11.4, 10.9, and 25.8 °C, respectively, which are close to the room temperature. The four transformation temperatures decreased slightly with length location of the sample increasing from top to bottom. Among them, *M*_s_ decreased from 7.0 to −5.0 °C, and *A*_f_ decreased from 25.8 to 4.0 °C. The decrement of the transformation temperatures along the length of the sample resulted from the composition change of the austenite matrix phase by the formation of the bainite phase, as mentioned in Sutou’s research [25]. The disappearance of the transformation peak intensity can be explained by the drastic decrement in the volume fraction of the austenite phase due to the formation of the bainite phase.

In order to study the change of shape memory properties of samples after the gradient heat treatment, samples #2 and #3 were selected to conduct compression experiments. Figure 5c shows the original width, the width after being compression loaded, the width after being unloaded, andthe width after being heated with the location along the length of the #2 sample. It can be seen that the graded dimension occurred both after being unloaded and heated. The #2 sample was compressed in the width direction by a deformation of 0.58 mm (~10%) and then unloaded. The sample could be recovered back to its normal width in a certain extent due to the elastic deformation and superelastic deformation, which, in this paper, were defined as elastic–superelastic strain (the value was equal to the sum of elastic strain and superelastic strain) to indirectly reflect the changes in superelastic properties. Due to the graded microstructure of the sample in the longitudinal direction, the elastic–superelastic strain also showed a gradient change. The elastic–superelastic strain (~8.5%) was the highest at the top of the sample with a low temperature heat treatment, and the elastic–superelastic strain (~2%) at the bottom with a high temperature heat treatment was the smallest. As mention above, the transformation temperature was close to room temperature. Though the stress-induced transformation occurred when compressed at room temperature, a large part of residual martensite in the alloy existed after unloading. Thus, after being unloaded, the sample was heated in boiling water at 100 °C for 10 min to cause the martensite reverse transformation of some residual martensite. It can be seen in Figure 5c that the loading strain in the width direction of the #2 sample after being water-boiled was further recovered, the total recovery strain (the value was equal to the sum of elastic–superelastic strain and water-boiled recovery strain) after being unloaded and water-boiled was also the maximum at the top of the sample (~9.7%), and the minimum was at the bottom (~3%), all of which show significant graded shape memory performance.

**Figure 6:**
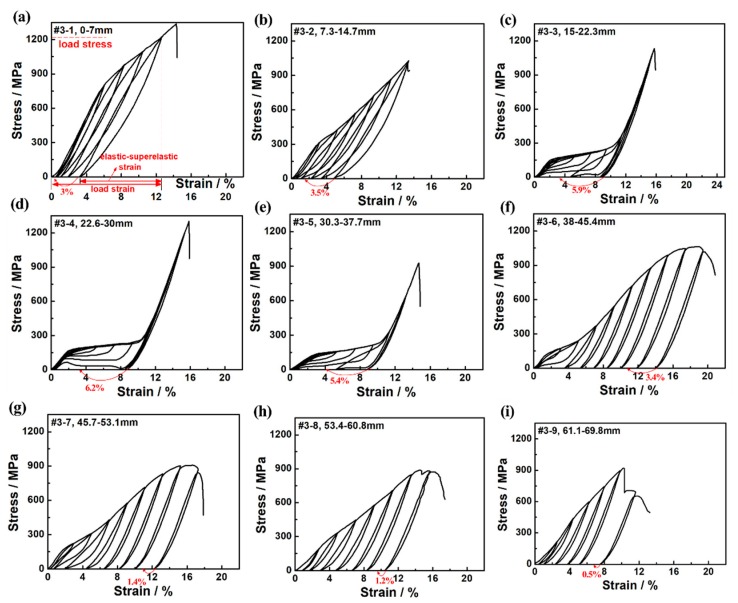
Compressive stress-strain cyclic loading-unloading curves of equal length regions from top to bottom of the #3 sample: (**a**) #3-1, 0~7 mm from the top; (**b**) #3-2, 7.3~14.7 mm from the top; (**c**) #3-3, 15~22.3 mm from the top; (**d**) #3-4, 22.6~30 mm from the top; (**e**) #3-5, 30.3~37.7 mm from the top; (**f**) #3-6, 38~45.4 mm from the top; (**g**) #3-7, 45.7~53.1 mm from the top; (**h**) #3-8, 53.4~60.8 mm from the top; and (**i**) #3-9, 61.1~69.8 mm from the top.

Figure 6a–i show the compression stress-strain cycle curves of nine regions from the top to the bottom of the #3 sample. It can be seen in Figure 6 that the curve shapes were obviously different in different regions. As the temperature of the sample increased from the top to the bottom, the elastic–superelastic strain first increased and then decreased gradually, while the residual strain first decreased and then increased gradually. In addition, the loading stress and fracture strength of different regions under the same strain also showed gradient changes.

**Figure 7:**
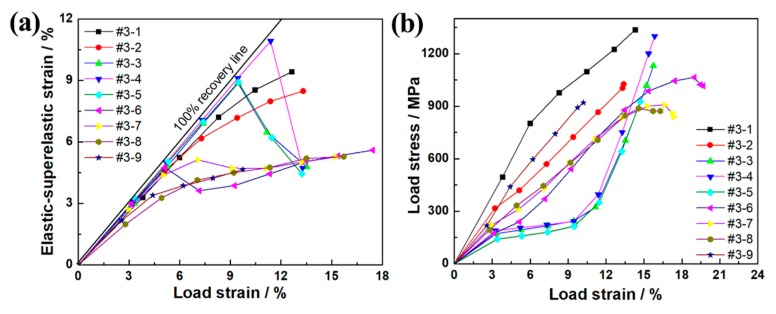
(**a**) Relationship between elastic–superelastic strain and loading strain and (**b**) relationship between loading stress and loading strain in different regions of sample #3.

In order to analyze the graded shape memory properties of the samples more intuitively, the relationship between the elastic–superelastic strain and the total loading strain can be obtained based on the data obtained from the stress-strain cycle curve in Figure 6. The differences in the elastic–superelastic strain property generated at different temperatures can be more visually compared, as shown in Figure 7a. The diagonal in the figure is the ideal 100% strain recovery line. The deviation between each curve and the ideal 100% line can directly compare the elastic–superelastic strain in each region of the #3 sample with the change of loading strain. As seen in Figure 7a, with the augment of loading strain, the recovery strain of the all regions basically showed a trend of increasing first and then decreasing, and the elastic–superelastic strain curve gradually deviated from the ideal 100% line. The inflection point of the elastic–superelastic strain curve was defined as the maximum elastic–superelastic strain. Meanwhile, the variation trend of recoverable strain in different regions of the sample can be seen in Figure 6a. The location of each region and the maximum elastic–superelastic strain are listed in Table 1. As seen in Table 1, the maximum elastic–superelastic strain changed from 10.9% to 4.6% from the top to the bottom of the sample, with a range of 2.4 times, which is consistent with the change of hardness.

It is worth noting that the strain recovery rates of #3-1 and #3-2 were worse than #3-3–#3-5 (as seen in Figure 6 and Table 1). This is because the DSC results mentioned above indicated that the martensite transformation temperatures at the regions where #3-1 and #3-2 were located were close to room temperature. When the superelastic property was tested at room temperature, a large part of the residual martensite phase after unloading existed. When the transformation temperatures of #3-3–#3-5 were lower than room temperature and there was no more bainite phase precipitation, the martensite reverse transformation could occur smoothly after being unloaded. After being unloaded, the nine regions in the #3 sample were also heated in boiling water at 100 °C for 10 min. The water-boiled recovery strains of all regions are marked with red arrows in Figure 6, and the values are listed in Table 1. It was found that after the water-boiled treatment, the compressive strain of the samples was further recovered about 0.5%~6.2%. As shown in Figure 6a, the extra water-boiled recovered strain of the #3-1 region reached 3%, which was equivalent to a total compression strain of 12.4% when added the elastic–superelastic strain.

Figure 7b shows the relationship between strain and stress under cyclic loading in each region. The loading stress corresponding to the end of each curve was the compressive strength of the region sample, which could reflect its resistance to failure. As can be seen in Figure 7b, from the top to the bottom of the sample, the number of loading cycles that #3-1–#3-9 could withstand increased gradually, and the compressive strength also showed a trend of gradual decrease. In Figure 6 and Figure 7b, the changes trend of the broken lines of #3-1–#3-9 are the same, which indicates that the change of the recovery strain of the sample was consistent with the change of the corresponding loading stress. The loading strain and loading stress corresponding to the maximum elastic–superelastic strain in Figure 7b are listed in Table 1. It can be seen that the loading stress corresponding to the maximum elastic–superelastic strain varied from the top to the bottom of the sample. Meanwhile, the loading strain corresponding to the maximum elastic–superelastic strain changed from 9.5% to 17.4%, with a change range of 1.8 times. The compressive strength, changing from 1337.2 to 887.3 MPa, had a change range of 1.5 times, and all the change rates showed a trend of first fast, then slow, and finally fast. Both graded bainite precipitated phase and transformation temperature change caused by the gradient heat treatment together affected the graded distribution of the strength level. The influence mechanism and control of these factors on the distribution of gradient stress are worthy of further research.

These results indicate that the shape memory properties and bearing capacity of the Cu–Al–Mn SMA change with gradient after a gradient heat treatment. In general, through a simple gradient treatment or a zone heat treatment, the devisable graded distribution of heat treatment temperature can be realized on the single component Cu–Al–Mn SMA, and the bainite phase shows a graded distribution to realize the customizable gradient of alloy hardness and shape memory properties.

## 4. Conclusions

In this paper, through a simple gradient heat treatment, the graded distribution of heat treatment temperature could be realized in the Cu–Al–Mn shape memory alloy, thus resulting in a graded distribution of the bainite phase to realize the gradient hardness and shape memory properties for this alloy. Some main conclusions are presented as followings:

(1) The amount and size of the acicular bainite precipitated phase of Cu_70.5_Al_19.5_Mn_10_ shape memory alloy can be changed by a gradient heat treatment. When the heat treatment temperature on the sample changes from 100 to 450 °C, the bainite precipitated phase produces from scratch and then grows continuously, finally presenting a graded distribution from one end section to another of the sample.
(2) After the gradient heat treatment, the Vickers hardness of the Cu_70.5_Al_19.5_Mn_10_ alloy samples presents a gradient change from the low temperature end to the high temperature end, from 215 to 388 Hv. There is a functional relationship between hardness *VH* and temperature *T*: *VH* = 390 − 170/[1 + (*T*/235)^18^].
(3) The transformation temperatures of the Cu_70.5_Al_19.5_Mn_10_ alloy decrease slightly with the increasing of heat treatment temperature. After the gradient heat treatment, the compression shape memory properties of alloy samples also show gradient changes from the low temperature end to the high temperature end. The elastic–superelastic strain shows a gradient change from 10.9% to 4.6%, with a change range of 2.4 times, while the compressive strength and water-boiled recovery strains also show a gradient change.

Overall, the gradient heat treatment can be used as an effective method for the gradient microstructure design or property control of Cu–Al–Mn alloy, and it is capable of providing important references for the function gradient treatment and practical application of this kind of high performance SMA material.

## Figures and Tables

**Table 1:** Maximum elastic–superelastic strain and corresponding loading strain and stress in different regions of the #3 sample.

Region Samples	Location from the Top/mm	Average Temperature/°C	Maximum Elastic–Superelastic Strain/%	Water-Boiled Recovery Strains/%	Corresponding Loading Strain/%	Corresponding Loading Stress/MPa	Compressive Strength/MPa
#3-1	0–7	98.5	9.4	3.0	12.6	1224.9	1337.2
#3-2	7.3–14.7	109.7	8.5	3.5	13.3	1004	1026.7
#3-3	15–22.3	135.7	8.8	5.9	9.6	245.7	1131.4
#3-4	22.6–30	146.3	10.9	6.2	11.4	395.7	1300.2
#3-5	30.3–37.7	160.9	8.9	5.4	9.5	214.8	926.7
#3-6	37–45.4	200.1	5.6	3.4	17.4	1045.5	1064.8
#3-7	45.7–53.1	226.1	5.3	1.4	15.2	900.2	908.4
#3-8	53.4–60.8	261.2	5.3	1.2	15.7	872.6	887.3
#3-9	61.1–69.8	360.9	4.6	0.5	9.74	892.2	920.5
